# Metabolic signatures accompany exploratory movement in climbing plants

**DOI:** 10.1371/journal.pone.0361761

**Published:** 2026-10-30

**Authors:** Valentina Simonetti, Silvia Guerra, Sara Avesani, Bianca Bonato, Marco Dadda, Laura Ravazzolo, Gabriela Gjinaj, Umberto Castiello

**Affiliations:** 1 Department of General Psychology, University of Padova, Padova, Italy; 2 Department of Agronomy, Food, Natural Resources, Animals and Environment (DAFNAE), University of Padova, Padova, Italy; University of California Riverside, UNITED STATES OF AMERICA

## Abstract

Climbing plants rely on exploratory movements to locate and reach supports in the environment. Circumnutation is central to this exploratory behavior, yet little is known about the physiological signaling dynamics that accompany this movement. Here we show that the presence or absence of a climbing support is associated with distinct volatile organic compounds (VOCs) intensity profiles in *Pisum sativum* plants. Using temporal analyses aligned with circumnutation cycles, we detected distinct VOCs patterns in plants growing with or without a potential support in the surrounding environment, even before physical contact occurs. Overall VOCs intensity and the coupling between VOCs signals and circumnutation movement descriptors were higher when supports were absent. This pattern suggests that support-seeking movements are associated with metabolic changes that are in line with early physiological adjustment associated with the environmental context. Our findings identify a previously undescribed association between VOCs dynamics and circumnutation modulation during support searching in climbing plants.

## Introduction

Climbing plants rely on exploratory growth movements to locate and secure mechanical supports, allowing them to reach light while minimizing structural investment [1,2]. A central component of this strategy is circumnutation, the rhythmic and often helical movement of growing organs such as the shoot apex [3]. Once regarded as a passive byproduct of growth, circumnutation is now recognized as a dynamic and adaptive process shaped by endogenous programs and environmental feedback [4–8]. Quantitative kinematic studies on *Pisum sativum* has shown that circumnutation sets the intrinsic pace of plant growth and functions as an exploratory behavior that enables climbing plants to progressively adjust growth trajectories until a suitable support is encountered [9,10].

Despite advances in understanding the biomechanics and developmental basis of circumnutation [5,11,12], the mechanisms through which climbing plants regulate and refine this exploratory movement remain poorly understood. Plant movements are tightly coupled to physiological and metabolic state [13], suggesting that volatile chemical signaling associated with metabolic activity may serve as a key clue in this process.

Volatile organic compounds (VOCs), known results of plant metabolic changes and mediators of plant-environment communication [14,15], represent a potential but largely unexplored link between plant metabolism and movement. VOCs participate in numerous physiological and ecological functions, including defense, pollinator attraction, and interplant signaling [16–23]. However, their relationship with mechanical behaviors, such as circumnutation, remains uncharted. We hypothesize that the environmental context defined by support availability modulates pea plants’ VOCs profiles and trends during exploratory behavior, prior to physical contact. We tested whether metabolic signaling is dynamically coupled to exploratory behavior under different environmental affordances. Specifically, we tested that (H1) support availability induces differentiation in VOCs profiles (differences in magnitude); (H2) VOCs exhibit support-dependent trends when aligned to circumnutation progression (differences in temporal dynamics); and (H3) VOCs-movement coordination differs by condition, consistent with support-dependent coupling between metabolic state and exploratory kinematics (differences in metabolic-kinematic coupling). H1 and H2 are logically independent. A VOC feature may show a constant intensity offset with no change in temporal trend (H1 only), a divergence that emerges or reverses over time but averages to near zero (H2 only), or both (H1 + H2). H3 considers movement as the reference variable, and therefore tests whether metabolic state couples with movement itself rather than time alone.

To investigate these hypotheses, we evaluated differences in VOCs profiles obtained using time-of-flight mass spectrometry (TOF-MS) between two different growth conditions (*No support*/*With support*), and we implemented a circumnutation-based analysis of VOCs intensity trends combining 3-dimentional (3D) motion reconstruction with real-time untargeted VOCs analysis. For this investigation we used *Pisum sativum* L., a model species with well-characterized circumnutation dynamics [10,24–26]. Using climate-controlled chambers, we monitored apex and tendril trajectories in 3D alongside VOCs intensities, in either the presence or absence of a support. This integrative approach allowed to explore, for the first time, whether volatile chemical signaling associated with metabolic shifts anticipates the support touch in climbing plants and whether this volatile signaling is coupled with the circumnutation progression during the support-reaching phase.

We show that the presence or absence of a support reshapes plant VOCs intensity and dynamics and produces distinct chemical signatures associated with circumnutation movements. Plants growing without a support exhibit higher VOC intensity and stronger coupling between VOC dynamics and circumnutation motion descriptors compared with plants growing with a support. These shifts highlight how environmental challenges can determine unique metabolic responses as a form of adaptive strategy. This underscores the critical role of environmental context in shaping plant anticipatory behavior and chemical signaling, offering new insights into how plants interact with their surroundings.

## Materials and methods

### Experimental design

This study was designed to investigate how the presence of a climbing support influences VOCs intensity profiles and circumnutation dynamics of pea plants (*Pisum sativum* L.). We established two growth conditions: (1) *With support*, in which plants were provided with a climbing structure (a wooden pole), and (2) *No support*, where plants grew without any support. The primary objectives were to test if (i) support availability induces differentiation in VOCs profiles, (ii) determine whether VOCs exhibit support-dependent trends when aligned to circumnutation progression, and (iii) assess coupling between VOCs intensity and circumnutation movement descriptors. VOCs were continuously measured using high-resolution MS with standardized sampling schedule, and plant 3D movement was collected using pair of cameras working in stereovision.

### Biological material and growth conditions

Snow pea (*Pisum sativum* L. var. saccharatum cv Carouby de Maussane; Fratelli Ingegnoli S.p.A., Milan, Italy) was selected as the study plant. Seeds were rinsed in tap water for 1 h, then germinated in darkness at 24°C (FOC 200IL Connected Cooled Incubator, VELP® SCIENTIFICA, Italy). Germination was done in filter paper strip (Whatman® paper 3MM Chr sheets, Cytiva, United States) soaked with demineralized water, with seeds placed 1.5 cm apart from each other and 0.5 cm from the top edge of the strip. Seeds were oriented with the hilum and micropyle facing downward. After four days of germination, seedlings were transplanted into the custom-built phytotron inside the Mind(the)Plant laboratory [27] that consists of 8 growth boxes, in which temperature, humidity, nutrient supply, light, and air recirculation are automatically managed (Fig 1). Each box has a volume of 1 m^3^ and houses a cylindrical pot (diameter 26.7 cm; height 20 cm) filled with silica sand (type 16SS, dimension 0.8–1.2 mm). An air treatment unit consisting of air prefilter and filter, fan, and steam humidifier (Esse Costruzioni S.r.l., Frosinone, Italy; Fig 1A), ensures the recirculation of purified air and the maintenance of set environmental parameters. A slight positive pressure (Δ 5 Pa from inside to outside) is ensured by continuous airflow.

**Fig 1:**
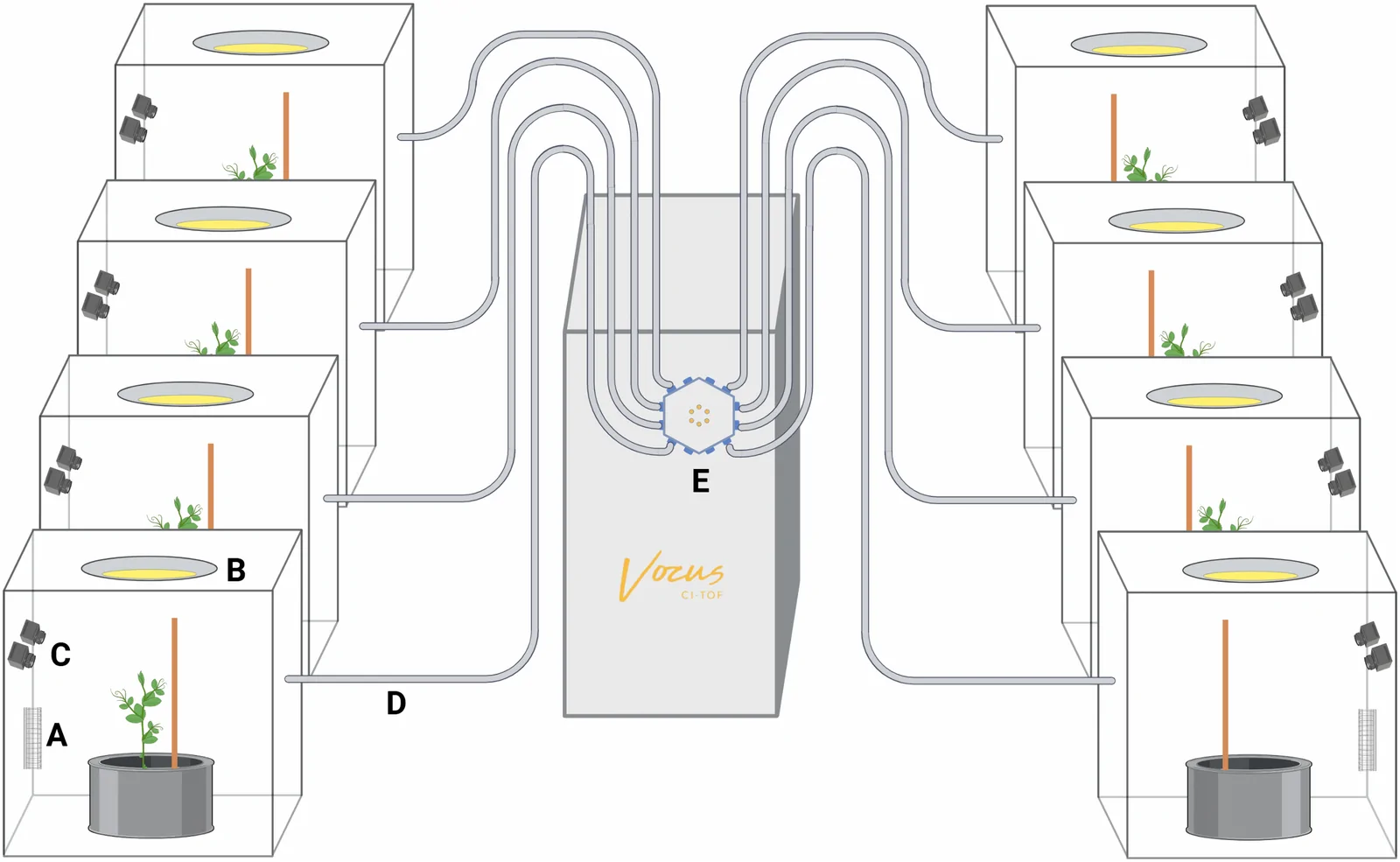
Experimental setup. Graphical representation of the experimental set up. (**A**) Air ventilation unit. (**B**) Cool white LED lamp (Q150W v2.0, PURE LED, Spain). (**C**) Pair of stereo cameras for simultaneous acquisition of plant images and 3D reconstruction of movement. (**D**) Perfluoroalkoxy (PFA) Teflon tubing (length: 6m; outer diameter 3/8 in). (**E**) Multiport valve (Tofwerk AG, Thun, Switzerland) mounted on the time-of-flight mass spectrometer (TOF-MS, Vocus 2R; Tofwerk AG, Thun, Switzerland). This image was made with BioRender (biorender.com).

One seedling was planted in each of the pots and grown for 15 days. All the boxes were planted simultaneously. To account for environmental differences across acquisitions, one growth box was left empty and considered as blank reference in the analyses. To maintain conditions similar, except for the presence of the plant, the box used as blank reference also contained the wooden pole (Fig 1). Additionally, the wooden pole was also present in the *No Support* condition, where it was placed in a corner of the box beyond the plant’s reach, thereby preventing physical interaction and attachment.

All pots were watered and fertilized every other day, using a half-strength solution culture (Murashige and Skoog Basal Salt Micronutriment Solution; 10 × , liquid, plant cell culture tested; SIGMA Life Science, Milan, Italy) with microfiltered water. Growth conditions were maintained at 24 ± 1.5 °C, 40 ± 10% relative humidity, and 11.15-hour photoperiod (5.45 am to 5 pm) under a cool white LED lamp (Q150W v2.0, PURE LED, Spain; Fig 1B) positioned 79.7 cm above each seedling. Photosynthetic Photon Flux Density of 200 umolph/(m2s) was measured at 79.7 cm under the lamp (HD2302.0 photometer with PAR LP471PAR probe, Delta Ohm, Germany) in correspondence of the seedling.

Two growth conditions were tested: (i) with a potential support in the environment (i.e., *With support*; Fig 2A) and (ii) without any support in the environment (i.e., *No support*; Fig 2B). The support used was a wooden pole (diameter 1.2 cm, total height 60 cm; 7 cm inserted into the substrate, 53 cm aboveground) positioned 10 cm from the seedling. Growth conditions (*No support*/*With support*) were alternated across series (“batteries”) of eight boxes, with the blank box randomly assigned in each battery. A total of 3 batteries per condition were performed obtaining data from 21 plants in the condition *With support* and 18 plants in the condition *No support* (data from 3 plants in the first battery were lost due to connection issues)*.* At the end of the 15 days of growth we collected morphological and weight measurements for each plant to assess overall well-being and population variability (descriptive statistics for morphological and weight data are available in S3 Dataset)

**Fig 2:**
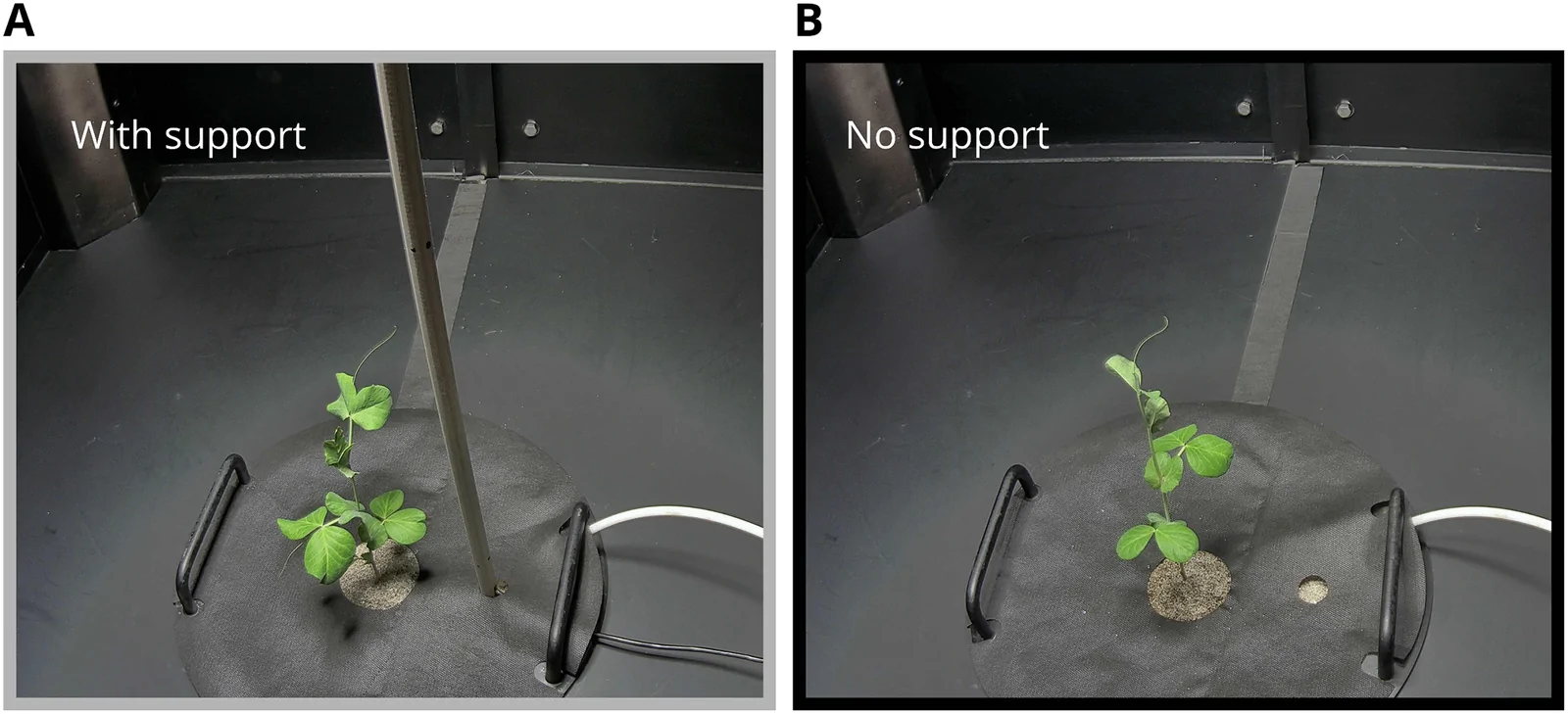
Experimental growth conditions. Pictures of the two growth conditions tested: (**A**) with a potential support in the environment (i.e., *With support*) and (**B**) without any support in the environment (i.e., *No support*).

### Kinematic data collection and movement descriptors

For each growth box, a pair of Power-over-Ethernet (PoE) 4K UHD RGB–infrared cameras (RLC-833A, Reolink; Fig 1C) was positioned 50 cm above the ground and spaced 45 cm apart to acquire stereo images of the plant. Cameras were connected via Ethernet cable to a router switch (UniFi Pro 24-Port, Ubiquiti Networks) on the same local network as the workstation used to manage the frames acquisition.

Camera calibration, frame acquisition and images storage process were managed using the custom software ROOMORS-CAMRECORDER (Ab.Acus s.r.l., Milan, Italy). Each camera was calibrated to estimate its intrinsic and extrinsic parameters using a 11  ×  8 chessboard pattern (square size: 20 mm) printed on a Forex® plate. Stereo calibration was then performed for each camera pair to determine relative positions, employing the same calibration pattern. Each pair of cameras was then programmed to acquire synchronously one frame every 3 minutes (frequency 0.0056 Hz).

Image sequences were processed using ROOMORS-TRACKING software (a graphical evolution of the SPROUTS software, Ab.Acus s.r.l., Milan, Italy [26]) to identify the anatomical landmarks on the plant and track their position frame by frame. The tracked points from both camera views were triangulated to reconstruct the 3D trajectories. All branches developed by the plants were tracked from the transplant until either the plant fell or coiled around the support. For all branches, the initial frame was defined as the first frame in which tendrils were visibly emerging from the apex.

The end of movement for the uncoiled branches was defined as the frame in which the tendrils stopped producing active circumnutation. For the last branch, the end of movement corresponds to the frame immediately preceding collapse or wrapping around the support.

The third branch of leaves was selected for the analysis, because it represented the branch of contact with the support in the condition *With support*. The anatomical landmarks analyzed were the apex and the tendril (Fig 3A). The apex is the tip of the shoot containing the apical meristem and it represents an index of growth of the plant, while the tendril is a modified leaf whose main function is to help the plant in the climbing process [28]. For each landmark, the 3D trajectory was reconstructed and processed to obtain circumnutation movement descriptors (Fig 3B, 3C). Following the computational approach described by Simonetti et al. [26] each circumnutation was isolated and the following movement descriptors were computed (Fig 3C):

- *Circumnutation length* [mm]: total 3D path length travelled by the landmark;
- *Circumnutation area* [mm^2^]: surface area enclosed in the 3D space, calculated by triangulating the circumnutation polygon relative to a reference vertex (the first point of the circumnutation) and summing the areas of the resulting triangles;
- *Circumnutation elongation*: ratio between the major axis of the circumnutation and the circumnutation length;
- *Circumnutation velocity* [mm/min]: mean velocity of the landmark along its 3D trajectory;
- *Circumnutation acceleration* [mm/min^2^]: mean acceleration of the landmark along its 3D trajectory;

**Fig 3:**
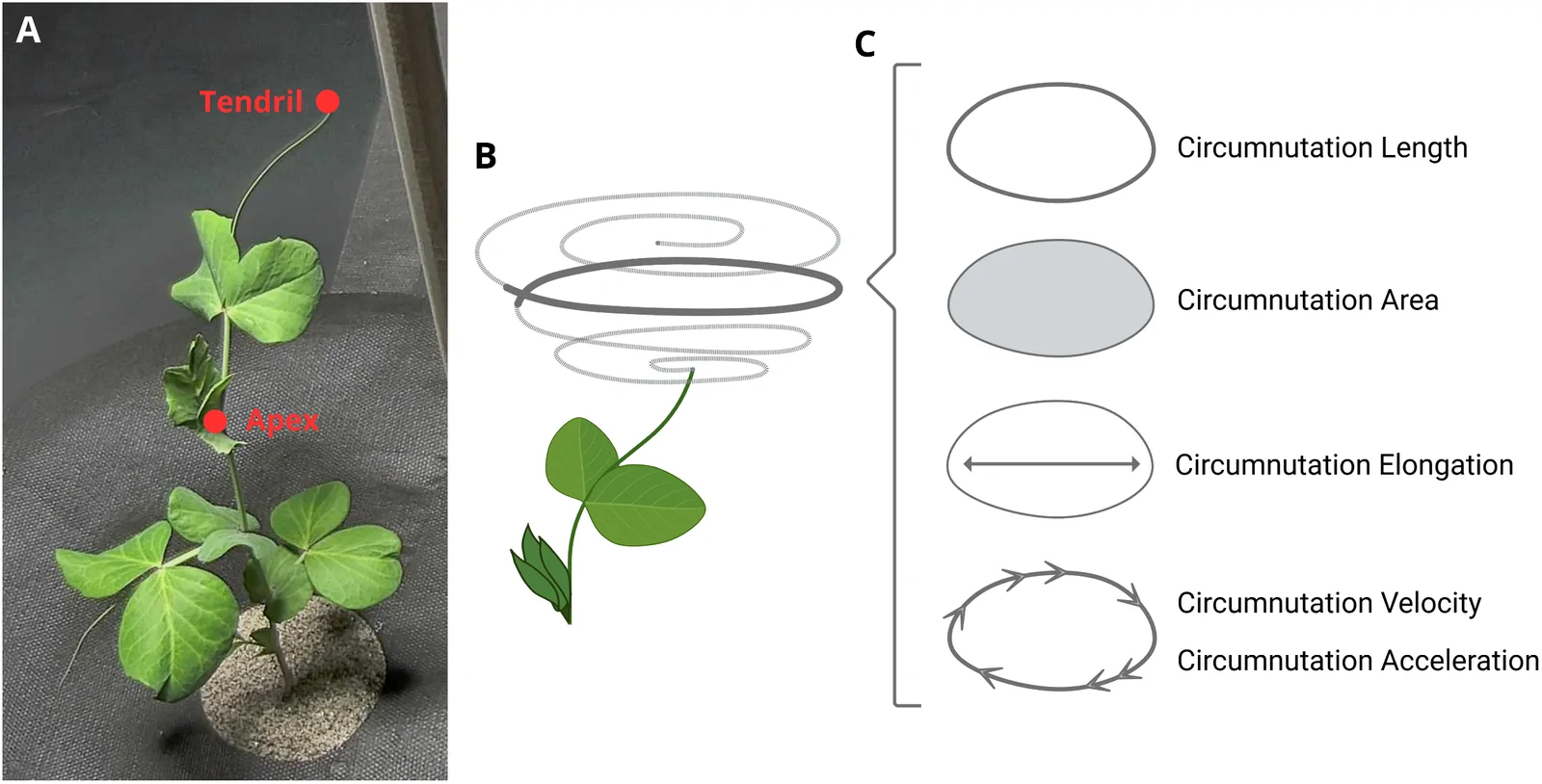
Circumnutation motion parameters. (**A**) Graphical representation on a sample pea plant of the anatomical landmark analyzed (in red): the apex and the tendril. (**B**) Graphical representation of the tendril movement trajectory where one single circumnutation is highlighted (dark gray). (**C**) Movement descriptors extracted from each circumnutation trajectory. This image was made with BioRender (biorender.com).

These movement descriptors were chosen because they provide a complete spatial and temporal description of the circumnutation movement that has shown to be crucial for the investigation of pea plant behavior [5,10,24,26].

### VOCs data collection and processing

VOCs were sampled from the growth boxes using perfluoroalkoxy (PFA) Teflon tubing (length: 6m; outer diameter 3/8 in; Fig 1D). A multiport valve (Tofwerk AG, Thun, Switzerland; Fig 1E) ensured the parallel connection of each tube to a time-of-flight mass spectrometer (TOF-MS, Vocus 2R; Tofwerk AG, Thun, Switzerland) equipped with an Adduct Ionization Mechanism (AIM) ion-molecule reactor operating in positive ionization mode with benzene as the reagent. VOCs were ionized in the Vocus AIM reactor, which combines a conductive polytetrafluoroethylene (cPTFE) conical reaction chamber, chosen for its fast response, with a differentially pumped, quadrupole-based interface to the TOF analyzer. Benzene was used as the reagent: gaseous benzene (≥ 99.9%; Sigma-Aldrich, St. Louis, MO, USA) was delivered from a permeation tube held at 80 °C into a vacuum ultraviolet (VUV) lamp housing, where photoionization produced C_6_H_6_^+^ reagent ions and photoelectrons. The reagent was supplied at 0.25 L/min, the AIM reactor was held at 50 °C, and the ionization source pressure was maintained at 50 mbar. Sensitivity and mass axis calibration were verified weekly using a mixture of benzene, toluene, m-xylene, α-pinene and 1,2,4-trimethylbenzene at 1 ppm, delivered at 0.01 L/min in ultra-high-purity N_2_. A detailed description of the operating condition and ionization parameters is reported in Avesani et al. [29].

The multiport valve was programmed to switch every 7 minutes (sampling rate: 0.5 Hz; flow rate: 2L/min), resulting in one acquisition cycle every 56 minutes for each growth box. In each battery, one growth box was randomly selected and left empty as environmental blank reference for VOCs analysis.

VOCs raw data were pre-processed using an untargeted metabolomics workflow in Tofware software (Version 3.2.5; Tofwerk) based on Igor software (Version 9.05 64-bit; WaveMetrics). The workflow comprised the following steps: (i) reference spectrum definition; (ii) peak width and shape refinement; (iii) mass calibration; (iv) peaks identification in a range of *m/z* from 30 and 250; and (v) data export. The resulting output consisted of .csv files containing data for each iteration (7 minutes acquisition with 0.5 Hz sampling rate) in which each row corresponds to a time point and each column represents the intensity of the detected signal. Files obtained were merged using a custom Python module (Python 3.12.3 version) in this way: for each 7-minutes iteration, the first 2 minutes and the last 1 minute were removed to account for the transition between growth boxes, then the average intensity value was computed. Merging all the iterations, we obtained a time series (one time point every 56 minutes) for each detected *m/z* signal for each growth box.

To manage instrumental and environmental variability, further processing steps were applied (custom Python module; Python 3.12.3 version). To account for fluctuations in reagent ion intensity each signal intensity at a given time point was normalized to the corresponding benzene ion intensity. In addition, to account for possible background environmental contributions, the signal intensity of each *m/z* channel was corrected by subtracting the corresponding intensity obtained from the empty box (blank reference) at each time point.

### Annotation

Molecular formulas of detected VOC signals were annotated using the online tool ChemCalc (https://www.chemcalc.org/mf-finder). Candidate formulas were compared, and the most probable chemical formula was selected based on the lowest mass error (≤ 10 ppm). Putative compound identities of molecular formulas of interest were inferred using the PubChem database (https://pubchem.ncbi.nlm.nih.gov/; [30]).

### Statistical analysis

#### Principal component analysis.

To evaluate the differences between the two growth conditions (*No support*/*With support*) we performed a PCA to reduce the dimensionality of the dataset and identify key patterns in the multivariate space. The features used for the PCA were the mean values of VOCs intensities detected. All the features were standardized using z-score normalization. The PCA was performed using Python scikit-learn library (version 1.5.1) function. To visualize the results obtained and identify global differences between conditions a 3D plot and a pairwise plot for the first three principal components were generated.

#### Supervised classification.

To complement the unsupervised PCA, we assessed whether the two growth conditions could be discriminated from the average VOCs intensity profiles using supervised models: regularized linear discriminant analysis (LDA) and random forest (RF) (Python scikit-learn library, version 1.8.0). For LDA, classification performance was estimated by stratified 5-fold cross-validation repeated 10 times, with feature standardization refitted within each training fold so that held-out samples did not contribute to preprocessing. Significance was assessed by a permutation test in which the condition labels were randomly reassigned 100 times and the entire cross-validation procedure was repeated. For comparison, the RF classifier (100 trees) was evaluated with the same cross-validation and permutation procedure.

#### Generalized additive mixed model for condition-dependent differences.

We analyzed each VOCs feature separately using a GAMM implemented in R using mgcv package (ban, fREML estimation). For each feature, we modeled the intensity as a smooth function of condition and circumnutation progression, including plant-level random effects to account for repeated measures. Specifically, we fit a GAMM with this structure:


$$\begin{array}{c} y_{ijt}=\;\beta_{\text{0}}+\;\beta_{\text{1}}Condition_{j}+f_{\text{0}}(t)+f_{\Delta }(t)Condition_{j}+b_{i}+f_{i}(t)+\varepsilon_{ijt} \end{array}$$ (1)

Where:

$y_{ijt}$ is the feature intensity for plant i, condition j, at time t

$\beta_{\text{1}}$ is the parametric condition effect

$f_{\text{0}}(t)$ is a smooth term modeling the population-level temporal trajectory

$f_{\Delta }(t)Condition_{j}$ is a condition-specific smooth for differences in trajectories

$b_{i}$ is a plant random intercept

$f_{i}(t)$ is a plant-specific smooth trajectory

$\varepsilon_{ijt}$ is the residual error

Analyses were restricted to the temporal window in which both conditions were represented by at least three plants per time point to ensure balanced support across conditions. We accounted for temporal autocorrelation using an AR(1) structure. The autocorrelation parameter (ρ) was estimated from model residuals within plants and aggregated across individuals using the median.

To test the first hypothesis (support availability induces differentiation in VOCs profiles; H1), we quantified the population-level predicted difference curve between conditions:


$$\begin{array}{c} \Delta (t)=y_{support}(t)-y_{no\;support}(t) \end{array}$$ (2)

Plant-specific terms were excluded to assess only population-level difference. Overall profile differentiation was summarized as the time-averaged difference between conditions. Statistical significance was evaluated by propagating model uncertainty through coefficient simulation. Coefficient vectors were sampled from the multivariate normal distribution defined by the estimated parameter vector and its covariance matrix. For each simulated coefficient vector, the time-averaged difference was recomputed, yielding a sampling distribution from which confidence intervals and two-sided p-values were computed. Resulting *p*-values were adjusted across all features using Benjamini–Hochberg False Discovery Rate (FDR). Significant *q* values indicate non-zero population-level difference between conditions.

To test the second hypothesis (VOCs exhibit support-dependent trends; H2) we evaluated the significance of the condition-by-time smooth term of equation (1). Significance was assessed using approximate smooth-term tests provided by mgcv, with FDR correction across all features. Significant *q* obtained indicates that the difference between conditions varies as a function of circumnutation progression, i.e., that VOC dynamics are support-dependent.

#### Generalized additive mixed model for metabolic–kinematic coupling.

To test whether the coordination between volatile emissions and exploratory movement differed across environmental contexts (H3), we extended the GAMM framework described above by incorporating kinematic descriptors of circumnutation as predictors of VOC intensity.

For each VOC feature and each kinematic variable, we fitted the following model:


$$\begin{array}{c} y_{ijt}=\;\beta_{\text{0}}+\;\beta_{\text{1}}Condition_{j}+f_{\text{0}}(t)+f_{\Delta }(t)Condition_{j}+g_{\text{0}}\left(k_{ijt}\right)+g_{\Delta }\left(k_{ijt}\right)Condition_{j}+b_{i}+f_{i}(t)+\varepsilon_{ijt} \end{array}$$ (3)

Where:

$y_{ijt}$ is the feature intensity for plant i, condition j, at time t

$\beta_{\text{1}}$ is the parametric condition effect

$f_{\text{0}}(t)$ is a smooth term modeling the population-level temporal trajectory

$f_{\Delta }(t)Condition_{j}$ is a condition-specific smooth for differences in trajectories

$k_{ijt}$ is the value of the kinematic variable measured at the same time point

${g_{\text{0}}(k}_{ijt})$ is a smooth function describing the shared coupling between VOC intensity and the kinematic variable

${g_{\Delta }(k}_{ijt})Condition_{j}$ is a condition-specific smooth allowing the VOC–kinematic coupling to differ between conditions

$b_{i}$ is a plant random intercept

$f_{i}(t)$ is a plant-specific smooth trajectory

$\varepsilon_{ijt}$ is the residual error

Kinematic variables were standardized prior to modeling to ensure comparable scaling of smooth terms. To evaluate whether metabolic–kinematic coupling differed between environmental contexts, we compared two nested models: a shared-coupling model including only ${g_{\text{0}}(k}_{ijt})$ and a differential-coupling model including both ${g_{\text{0}}(k}_{ijt})$ and ${g_{\Delta }(k}_{ijt})Condition_{j}$. Evidence for condition-dependent coupling was assessed using likelihood ratio tests between these nested models. This procedure was repeated for all combinations of VOC features and kinematic descriptors of circumnutation. Resulting p-values were corrected for multiple comparisons using the Benjamini–Hochberg false discovery rate procedure. All models were fitted using the mgcv package in R with fREML estimation and plant-level random smooths to account for repeated measurements and individual growth trajectories.

In addition to testing for condition-dependent differences in coupling (H3), we evaluated whether significant metabolic–kinematic coupling was present within each growth condition independently. For each VOC feature and kinematic variable, we fitted condition-specific GAMMs including plant-level random effects and temporal smooths, and compared a baseline model including only temporal dynamics and plant-level structure to a model additionally including a smooth term for the kinematic predictor. Evidence for coupling within a condition was assessed using likelihood ratio tests between these nested models. This procedure was repeated for all VOC–kinematic combinations, and all the resulting *p*-values were corrected using FDR.

## Results

### Support availability influences VOCs magnitude and temporal dynamics

Principal component analysis (PCA) was performed to evaluate global differences in average VOCs intensities between the two growth conditions (*No support*/*With support*; Fig 4A). The two growth conditions were largely separable in the space of the first three principal components, indicating that the presence or absence of a potential support in the environment influences the average VOCs intensity profiles (Fig 4B, 4C).

**Fig 4:**
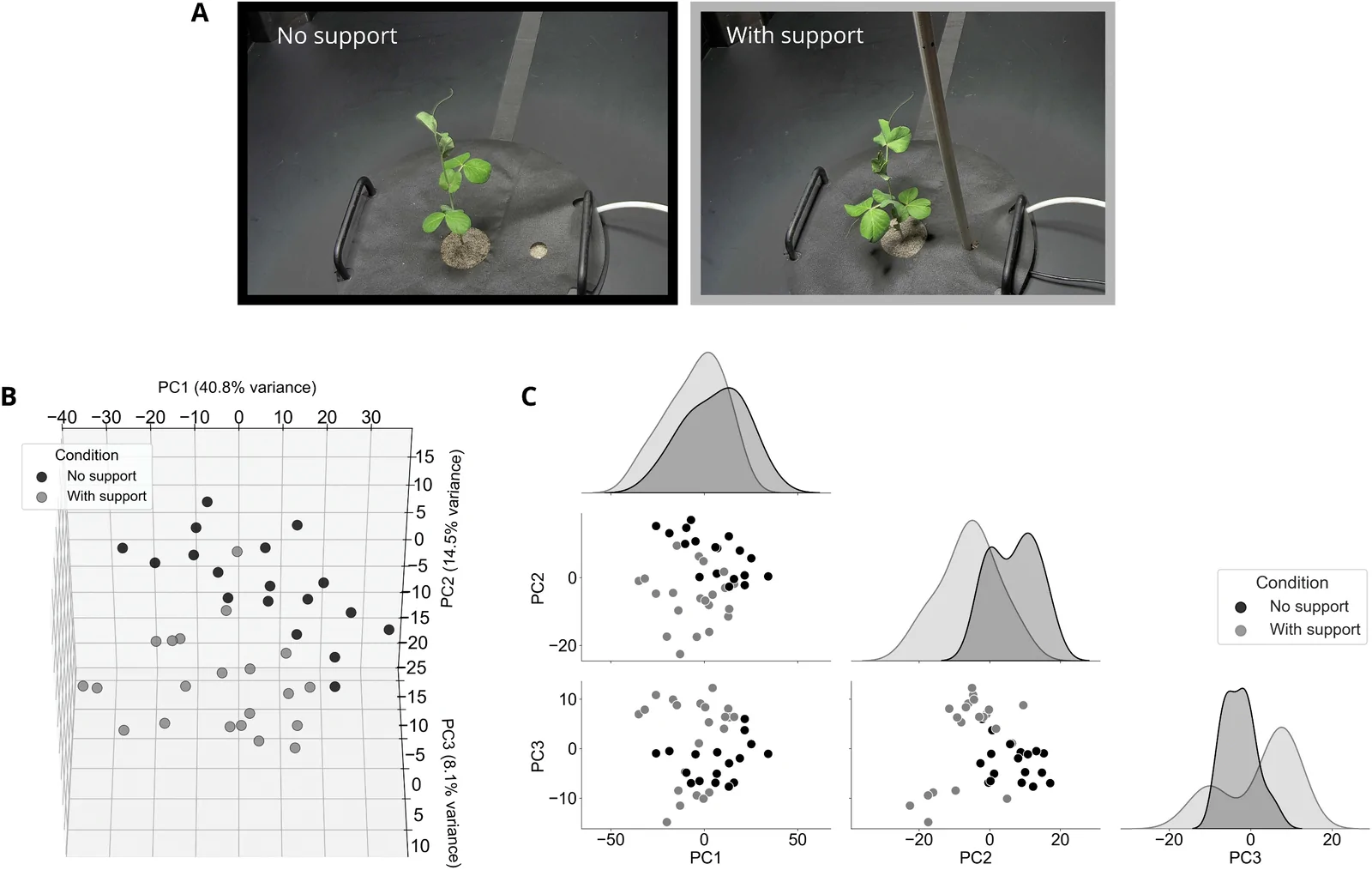
Results of PCA for different growth conditions. (**A**) Sample pictures of plants for the two growth conditions (*No support*/*With support*). (**B**) Result of the PCA obtained comparing the average values of VOCs intensities in the two growth conditions. (**C**) Pairwise representation of the samples on the first three principal components obtained with the PCA.

Consistent with PCA results, supervised classification of the same features distinguished the two growth conditions with a cross-validated accuracy of 0.91 ± 0.07 (SD) for regularized linear discriminant analysis and 0.90 ± 0.09 (SD) for a random forest classifier, against a majority-class baseline of 0.54. Under random permutation of the condition labels, accuracy fell to 0.50 ± 0.09 and 0.50 ± 0.10 respectively (p < 0.01, 100 permutations).

A generalized additive mixed model (GAMM) including a condition effect, a population-level smooth term, condition-specific smooth deviation, and single plant as random intercept was implemented to model the progression of signal intensity for each detected VOCs feature. This model was used to test the first hypothesis (support availability induces differences in VOCs magnitude; H1) and the second hypothesis (support availability induces differences in VOCs temporal dynamics; H2). To test H1, we quantified the time-averaged predicted population difference between conditions and assessed significance by propagating model uncertainty through simulations from the coefficient covariance matrix, generating confidence intervals and two-sided *p*-values. Resulting *p*-values (*p*_*avgdiff*_) were corrected using False Discovery Rate (FDR) obtaining *q*_*avgdiff*_. To test H2, we evaluated the significance of the condition-specific smooth term of model assessing the significance using approximate smooth-term tests. Resulting *p*-values (*p*_*traj*_) were corrected using FDR obtaining *q*_*traj*_. A significant *q*_*traj*_ indicates that temporal dynamic for that VOCs feature is support-dependent. 219 compounds showed a support-dependent difference in magnitude (H1; *q*_*avgdiff*_ <0.05) and 68 showed support-dependent temporal dynamics (H2; *q*_*traj*_ <0.05) and from the volcano-style plot of the global profile differentiation (Fig 5A) we can see that the condition *No support* reports higher number of compounds with greater intensity compared to the condition *With support*. From the volcano-style plot of the support-dependent temporal dynamics (Fig 5B) we can see that most of the compounds with significant difference in temporal dynamics between conditions are also compounds with significant global profile differentiation (H1 + H2) (complete set of compounds details is available in S1 Dataset). The same features are shown across the mass axis in Fig 5C, where the difference in mean normalized signal between conditions is displayed at each detected *m/z*. Effect sizes are reported for every feature in S1 Dataset, which lists the time-averaged population-level difference between conditions with its 95% confidence interval, the maximum absolute predicted difference across the trajectory, and the deviance explained. Fig 6 shows some sample VOCs differences in predicted trajectories between growth conditions, selected amongst the most significant.

**Fig 5:**
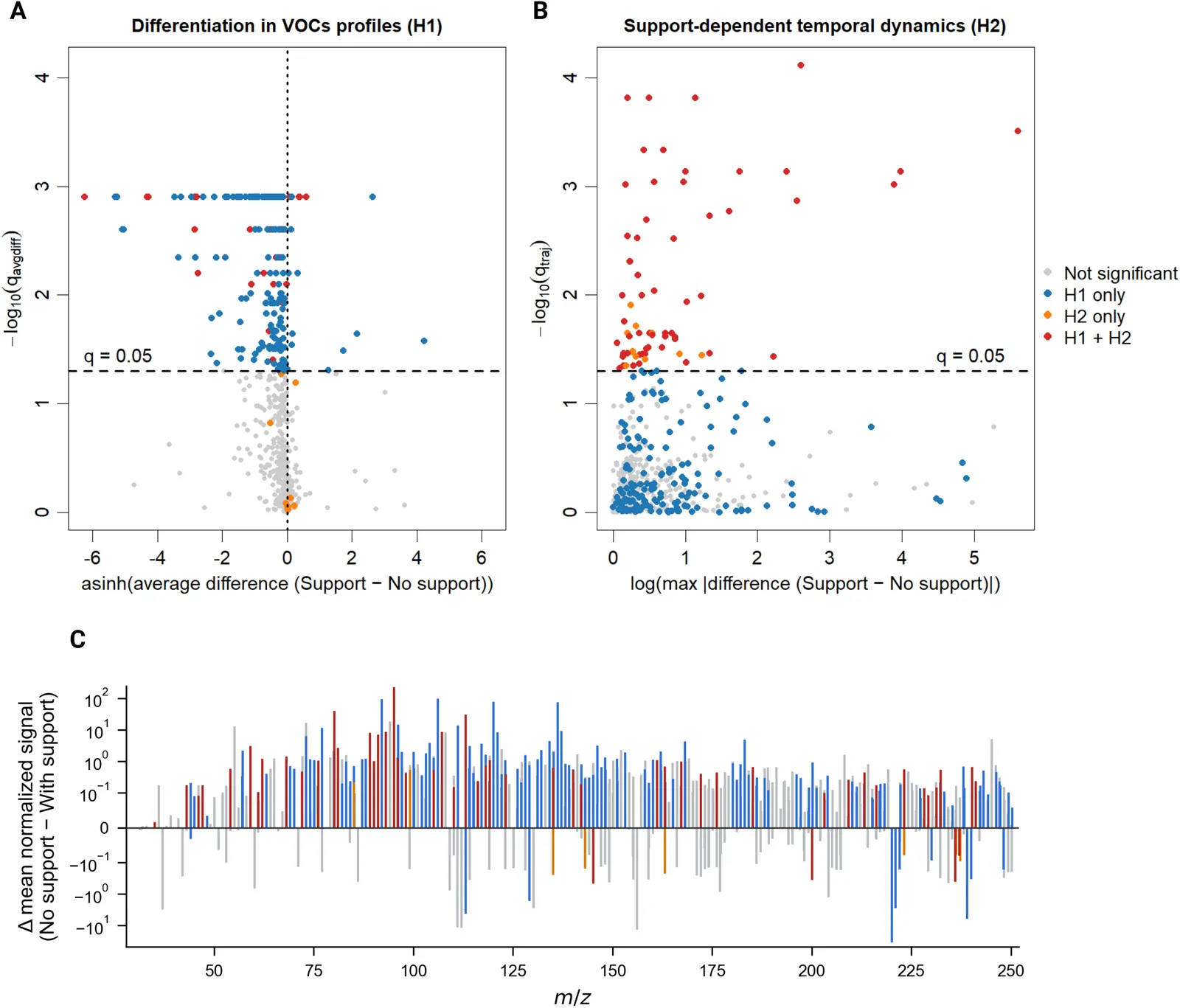
Metabolic profile differentiation and support-dependent temporal dynamics of VOCs. Volcano-style plots and spectrum summarizing the global differentiation in VOCs magnitude (H1) and differences in temporal dynamics (H2). Each point in the volcano-style plots and each line in the spectrum represents one VOC feature, colored according to its significance: non-significant (grey), significant for H1 only (blue), significant for H2 only (orange), and significant for both hypotheses (red). The horizontal dashed line in the volcano-style plots indicates the significance threshold (*q* = 0.05). (**A**) Global profile differentiation in magnitude between experimental conditions (H1). The x-axis represents the time-averaged population-level difference between growth conditions. Values are shown using an inverse hyperbolic sine transformation to improve visualization while preserving sign and relative magnitude. The y-axis shows the statistical significance. (**B**) Support-dependent temporal dynamics of VOCs intensity (H2). The x-axis represents the maximum absolute predicted difference between conditions across the temporal trajectory, summarizing the magnitude of condition-dependent deviations in the VOC trajectory. Values are log-transformed for visualization. The y-axis shows the statistical significance of the condition-specific smooth term of the GAMM. **(C)** Difference spectrum across the analyzed mass range. Each vertical line represents one VOC feature, positioned at its m/z and drawn at the difference in mean normalized signal between growth conditions (No support – With support). Values were averaged within each plant and then across plants. The vertical axis uses a symmetric logarithmic scale, linear between −0.1 and 0.1.

**Fig 6:**
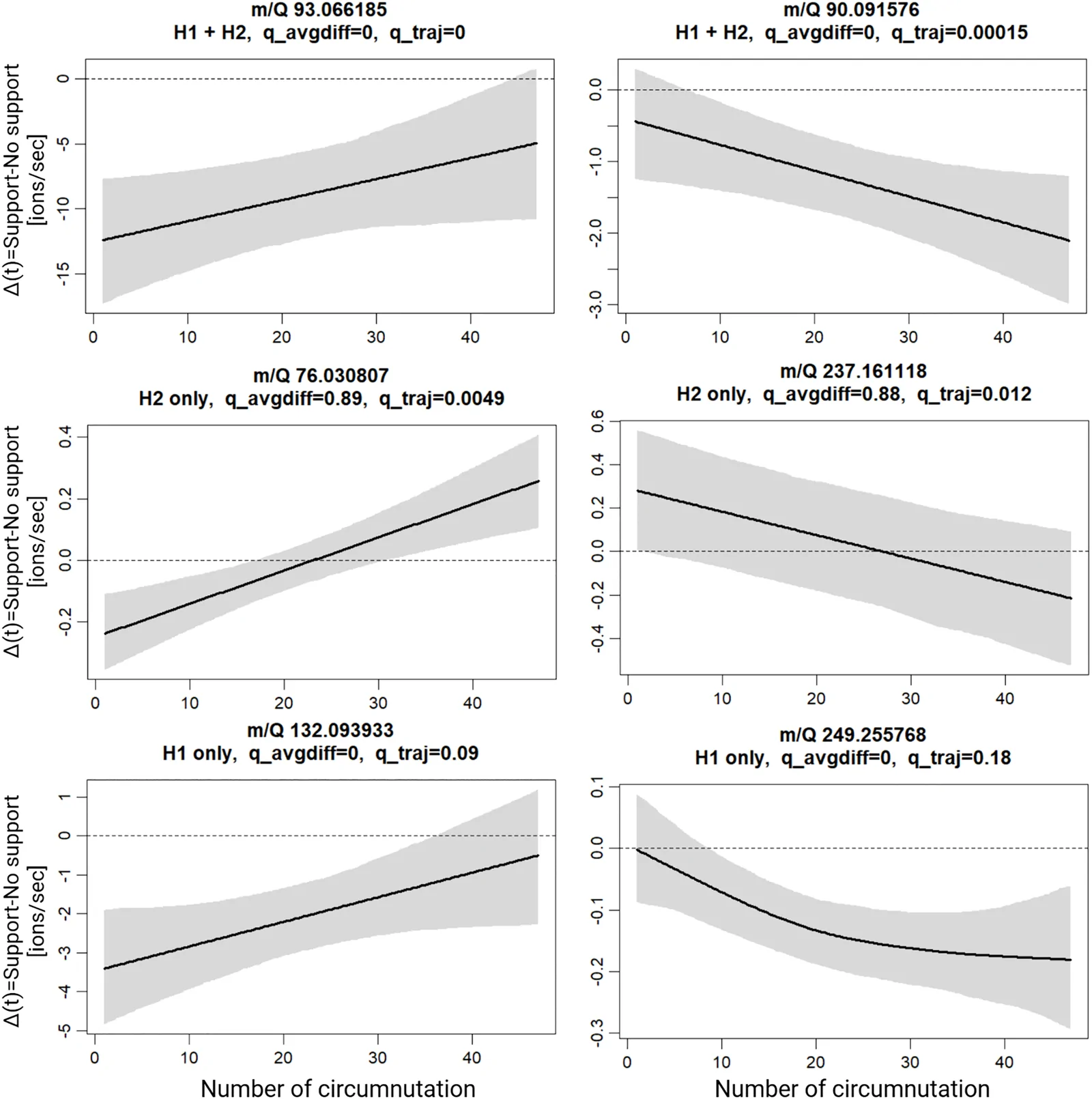
Predicted difference curves between growth conditions for representative VOC features. Each panel shows the population-level difference in predicted signal intensity over time, obtained from the fitted GAMM. The black line represents the estimated difference curve, while the shaded grey band indicates the 95% confidence interval derived from the model covariance matrix. Panels are grouped according to the statistical outcome of the two tests: VOC features showing both a significant overall profile shift and support-dependent temporal dynamics (H1 + H2), features exhibiting only dynamic differences over time (H2 only), and features showing a consistent overall shift between conditions without significant trajectory modulation (H1 only).

We performed preliminary contextualization of biologically relevant mass features by evaluating whether the detected mass features showed compatible putative molecular formulas with VOCs previously reported in *Pisum sativum* [16]. From this explorative annotation of some of the detected masses showing significant profile differentiation or support-dependent temporal we found correspondences with five compounds identified in previous studies on *Pisum sativum* [31–35] (Table 1). All the 5 compounds identified show a global profile differentiation between growth conditions and no significant difference in temporal dynamics, which means that the compounds have overall different intensity level but no difference in their trend.

**Table 1:** Detected compounds with correspondence in literature. Correspondence between putative molecular formulas of detected compounds showing significant differences between growth conditions and annotated VOCs from other studies on Pisum sativum. The table reports the compounds with at least one correspondence with previous studies. Studies included were both on constitutive VOCs and stress-induced VOCs.

Molecular formula	Monoisotopic mass	Detected mass	Annotated VOCs	Growth conditions	Type	Ref.
C10H12O	*m/z* 148.08882	*m/z* 148.088867	6-allyl-o-cresol	potted living plants	constitutive	[35]
C10H16	*m/z* 136.1252	*m/z* 136.124634	limonene, D-limonene, α-fenchene, 3-carene, camphene, myrcene, terpinolene, α-pinene, β-pinene, terpinene, β-ocimene, (E)-β-ocimene, (Z)-β-ocimene, α-phellandrene	potted living plants, vegetative stage, flower stage, pod formation stage, leaf emission, bud emission, flower emission, aphids, virus and aphid	constitutive, stress-induced	[31–35]
C8H10	*m/z* 106.07825	*m/z* 106.077713	ethylbenzene, o-xylene, p-xylene, m-xylene	potted living plants, aphids	constitutive, stress-induced	[35]
C7H8	*m/z* 92.0626	*m/z* 92.062126	toluene	bud emission	constitutive	[33]
C11H16	*m/z* 148.1252	*m/z* 148.124466	1-methylbutyl-benzene	vegetative stage, flower stage	constitutive	[34]

### Condition-dependent coupling between VOCs profiles and circumnutation

To test the third hypothesis (VOCs-movement coordination differs by condition; H3), we extended the GAMM used in the previous section by incorporating kinematic descriptors of circumnutation as predictors of VOCs intensity. Throughout, “coupling” denotes a statistical association between VOCs intensity and a kinematic descriptor within the model, without implying a causal or mechanistic link. We evaluated whether significant metabolic-kinematic coupling was present within each growth condition independently and we tested for condition-dependent differences in coupling. We performed the same analysis both for kinematic parameters of the apex and the tendril (Fig 3). Globally, we obtained that more VOCs are significantly coupled with tendril kinematics than with apex kinematics in both the growth conditions and that they show higher differential coupling with tendril kinematics than apex kinematics (Fig 7A and 7B). For almost all the kinematic parameters, more VOCs are significantly coupled in the condition *No support* than the condition *With support* (Fig 7C and 7D) (complete set of compounds details is available in S2 Dataset). Fig 8 shows the relationship between sample VOCs features (those significantly coupled for both growth conditions) and tendril circumnutation motion parameters. Effect sizes for the coupling analyses are reported in S2 Dataset, which lists for every VOC-kinematic pair the root-mean-square difference between the condition-specific coupling functions, the difference in AIC between the nested models, and the deviance explained. Among the tendril pairs showing significant condition-dependent coupling, the deviance explained had a median of 0.17 (IQR 0.10–0.39).

**Fig 7:**
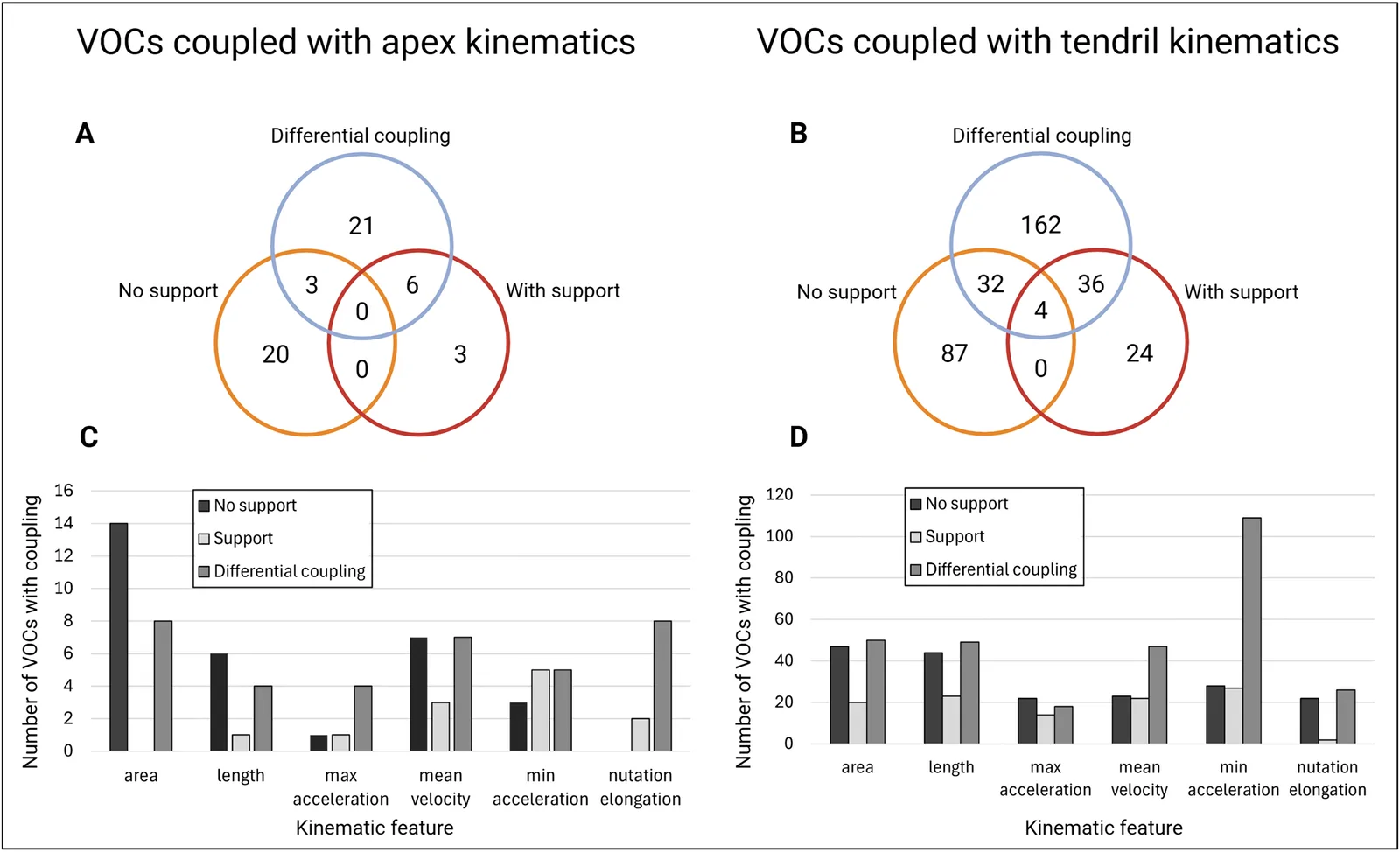
Metabolic–kinematic coupling between VOC emissions and circumnutation dynamics in apex and tendrils. (**A–B**) Venn diagrams showing the number of distinct volatile organic compounds (VOCs) significantly coupled with kinematic variables under different growth conditions: *No support* (orange), *With support* (red), and VOCs whose coupling with kinematic variables differed significantly between conditions (blue). (**A**) Results for apex kinematics and (**B**) for tendril kinematics. (**C–D**) Bar plots summarizing the number of VOC features significantly associated with individual kinematic descriptors of circumnutation: area, length, maximum acceleration, minimum acceleration, mean velocity, and nutation elongation. Bars represent couplings detected in the *No support* condition (dark gray), *With support* condition (light gray), and VOC-kinematic relationships that differed significantly between environmental contexts (medium gray). (**C**) Bar plot for apex kinematics and (**D**) for tendril kinematics.

**Fig 8:**
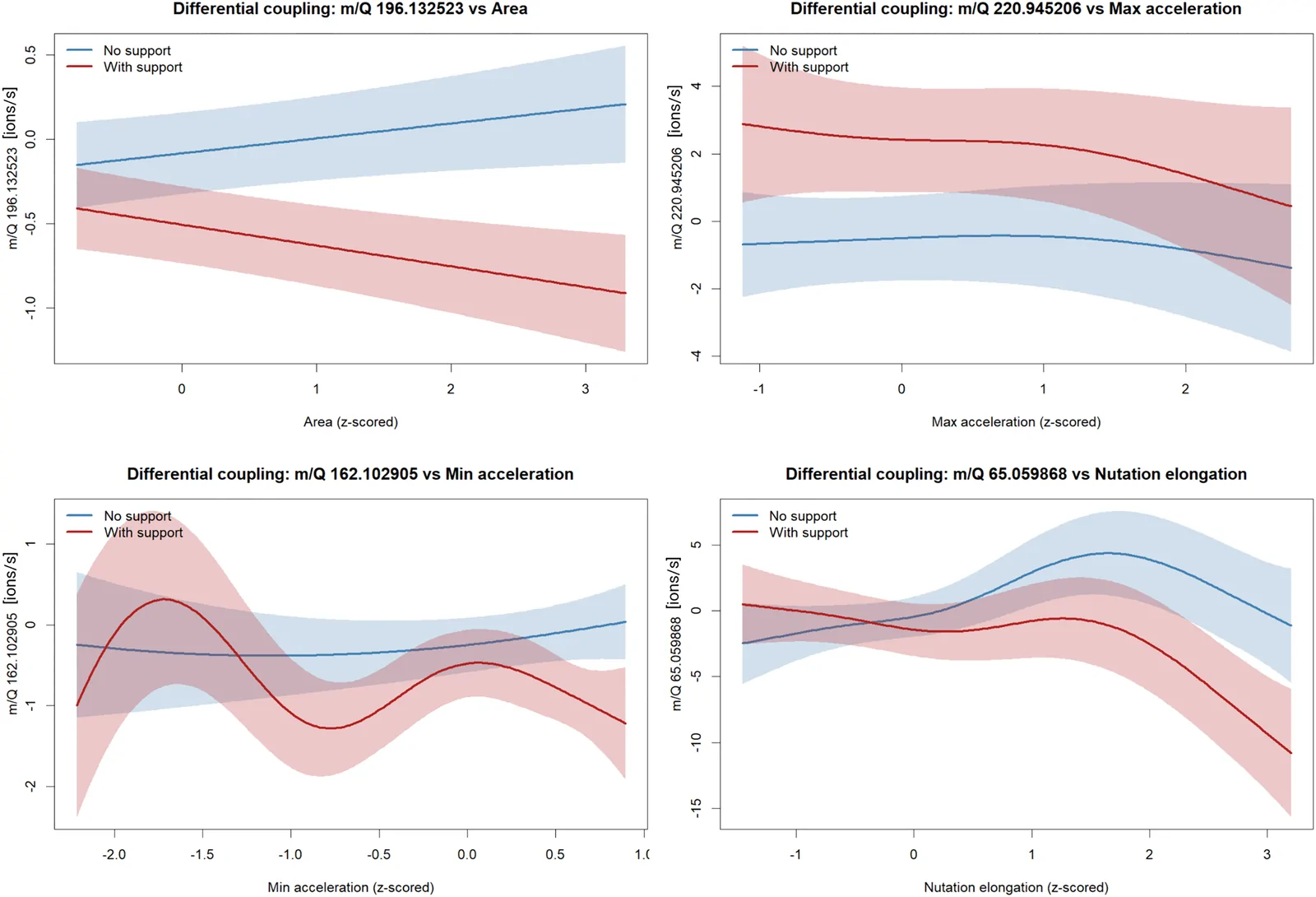
Differential coupling between VOCs intensity and circumnutation descriptor. GAMM fits showing the relationship between sample VOCs features and tendril circumnutation motion parameters. The relationships reported are those significant in both the growth conditions and with differential coupling. Solid lines represent the estimated smooth functions from the GAMM, and shaded areas indicate ±1.96 standard errors.

## Discussion

Our results indicate that the presence or absence of a climbing support significantly alters the detected VOCs profile of pea plants at the three levels considered: magnitude (H1), temporal dynamics aligned with circumnutation progression (H2), and coupling with movement descriptors (H3). The primary contribution of the present study is not compound-level identification, but the demonstration that support availability is associated with condition-dependent VOCs dynamics temporally linked to exploratory movement. This differentiation in VOCs profiles takes place before physical contact with the support indicating that environmental context influences the plant’s physiological state during the exploratory phase of growth and that metabolic shifts observed are not exclusively induced by thigmotropic responses in the classic vision on tendril support-seeking and reaching behavior [36]. Such sensitivity is consistent with growing evidence that circumnutation is an adaptive behavior modulated by environmental information rather than a passive by‑product of growth [7,10,37].

At the level of VOCs magnitude (H1), plants growing without a support exhibited higher overall VOCs intensity than plants provided with a potential support. This pattern potentially indicates that plants growing without a support exhibit distinct metabolic dynamics associated with the environmental context. VOCs are known to reflect metabolic shifts linked to environmental challenges and plant communication [17,20,21]. In this context, increased VOC output may represent a metabolic signature accompanying the plant’s search for structural support.

Beyond this overall offset, temporal analyses aligned with circumnutation cycles (H2) revealed that a subset of VOCs displays support‑dependent trajectories. Because these differences arise before contact with the support, they suggest that plants change their metabolic activity during exploratory growth rather than only in response to mechanical stimulation. These findings extend previous kinematic studies showing that climbing plants modify their movement dynamics in anticipation of environmental opportunities [10,25,37]. The mechanism through which climbing plants detect a potential support prior to contact is still under investigation. Different non-mutually exclusive possibilities may account for this anticipatory adjustment [1] including sensitivity to environmental cues such as light gradients [38–40], changes in VOCs composition or concentration [41], and prior root-mediated interactions [42]. Relative humidity and air flow were regulated and controlled inside the box, so all the boxes had the same global condition. Nevertheless, a rigid object positioned 10 cm from the plant can locally perturb the boundary layer around the shoot and tendrils and may generate microscale gradients in air movement and humidity. Among these possibilities, airborne chemical cues and microenvironmental light asymmetries appear particularly compatible with the anticipatory nature of the observed responses. We cannot exclude that multiple mechanisms coexist simultaneously and that their relative contribution differs across plant species. Although the present study was not designed to isolate these mechanisms, future experiments following this line of investigation could compare supports of different materials, transiently present or remove supports, or expose plants to support-derived material without mechanical affordance. This will help to disentangle the relative contribution of chemical and physical cues in pre-contact support perception.

Turning to movement as the reference variable (H3), the metabolic-kinematic analysis indicates that VOC emissions are more strongly associated with tendril movement than with apex motion. Tendrils represent the plant organ specialized for environmental probing and attachment [28,43]. The stronger coupling between VOCs intensity and tendril dynamics may reflect the functional specialization of tendrils in the exploratory behavior. This suggests that metabolic adjustments may accompany the plant structures most directly involved in spatial exploration and support reaching, or that the biomechanical characteristics of the tendril, including greater movement amplitude and spatial dynamics, may contribute to the stronger coupling observed.

One possible explanation for the observed differences between growth conditions is that both VOCs profiles and circumnutation kinematics are jointly regulated by broader physiological mechanisms associated with support availability. While still under investigation, circumnutation seems to depend on endogenous growth-regulating processes involving hormonal signaling and differential growth [5,44], suggesting that the observed metabolic-kinematic coordination may reflect integrated physiological adjustment during exploratory growth. In this context, lignin-related metabolism may represent one possible contributing pathway. Because lignin biosynthesis is tightly linked to the mechanical requirements of plant growth and self-support [45], some of the metabolic differences observed between plants growing with or without a climbing support may reflect differences in structural investment. Future studies combining real-time VOCs monitoring with lignin staining and transcriptomic analyses could help clarify the relationship between exploratory behavior, VOCs dynamics, and structural metabolism in climbing plants. On the signaling side, quantifying hormones associated with growth and stress responses alongside VOCs would connect the volatile signal to the physiological pathways that regulate circumnutation.

Although the molecular identity of most detected compounds remains provisional, some detected masses find correspondence with compounds previously reported in *Pisum sativum* [31–35] (Table 1). These correspondences situate the observed VOCs profile variation within known chemical repertoires of pea plants while highlighting a previously unexplored link between VOCs dynamics, environmental context and plastic growth. Among the formulas with correspondence in previous studies, C_10_H_16_ is one of the most interesting biologically. This formula corresponds to the monoterpenes which are among the best characterized plant VOCs and contribute to different plant functions such as defense against herbivores and pathogens, responses to stress and plant-plant signaling [16,20,21]. Their higher intensity in plants growing without a support is compatible with a broader shift in secondary metabolism accompanying exploratory growth.

Some limitations should be considered. Compound identification is based on high‑resolution mass measurements and therefore remains putative without structural confirmation using complementary analytical approaches such as GC-MS or tandem MS. In particular, exact mass information alone does not allow confident discrimination among structurally related compounds or isomers. In addition, untargeted real-time metabolomics approaches may preferentially detect abundant or highly ionizable compounds, which are not necessarily those with the greatest functional relevance. Finally, it is important to further stress that the present analyses reveal correlations between VOCs emissions and circumnutation but do not establish causal relationships between metabolic signaling and movement. The current data do not allow us to determine whether VOCs modulation influences circumnutation, whether movement dynamics affect VOCs profiles, or whether both processes are jointly regulated by upstream physiological mechanisms associated with environmental perception. Nevertheless, our results show that circumnutation dynamics and VOCs modulation exhibit related patterns under different environmental conditions, indicating metabolic-kinematic responses associated with support availability.

## Supporting information

S1 Text Sensitivity analysis. Supporting information showing the detailed methodology and results of the sensitivity analysis excluding outlier plants.
(DOCX)

S1 Movie Timelapse of a sample plant growing in the condition *No support.* (MP4)

S2 Movie Timelapse of a sample plant growing in the condition *With support.* (MP4)

S1 Dataset Results from the generalized additive mixed model (GAMM) testing differences between conditions (*With support* VS *No support*) over time for each feature (H1 and H2). Significance of the average predicted difference between conditions across the supported time window is reported as q_avgdiff and significance of trajectory differences between conditions is reported as q_traj. *P*-values were adjusted for multiple testing using Benjamini-Hochberg false discovery rate (FDR). avg_diff represents the mean predicted difference between conditions across the supported time window, with corresponding confidence intervals (avgdiff_ci_low, avgdiff_ci_high), while max_abs_diff reports the maximum absolute predicted difference between conditions across time, dev_exp is the proportion of deviance explained by the model and r_sq is the adjusted R-squared of the fitted model. The table also reports model complexity metrics including the effective degrees of freedom of the time smooth (edf_time), the condition-specific trajectory difference smooth (edf_diff), and the plant-specific factor smooth (edf_fs).
(XLSX)

S2 Dataset Results from GAMM testing associations between each VOC feature and each kinematic variable. Coupling between VOC intensity and kinematic variables was tested separately within the conditions and for differences in coupling between conditions. Raw *p*-values are reported for each test, and corresponding FDR-adjusted *p*-values are provided as *q*-values. The magnitude of the difference between condition-specific coupling curves is summarized by rms_diff, representing the root mean square difference between predicted coupling functions across the overlapping kinematic range. delta_aic is the difference in AIC (Akaike Information Criterion) between coupling models, dev_exp is the proportion of deviance explained by the model and r_sq is the adjusted R-squared of the fitted model.
(XLSX)

S3 Dataset Descriptive statistics for morphological parameters and physiological parameters collected with dualex. Both morphology and dualex data have been collected at the end of each experiment.
(XLSX)
