# Supplementary material for: Metabolic signatures accompany exploratory movement in climbing plants
Source: PLoS One. 2026 Oct 30;21(10):e0361761. doi: 10.1371/journal.pone.0361761 (PMC13722212; doi:10.1371/journal.pone.0361761)
Supplement: Supplementary file 1 [file pone.0361761.s001.docx]

**Supporting Information for:**

**Metabolic signatures accompany exploratory movement in climbing plants**

**Sensitivity Analysis**

To further assess the robustness of the main condition-dependent VOCs modulation patterns, we performed a sensitivity analysis excluding plants with extreme global VOCs profiles. Importantly, all GAMMs used in the present study already explicitly account for individual-level variability through plant-level random effects, plant-specific temporal smooths, and autocorrelation structures. The sensitivity analysis was therefore intended as an additional robustness assessment rather than as a correction for unmodeled variability.

Outlier plants were identified independently from the statistical significance of the GAMM results. For each plant, a global mean VOCs intensity value was calculated across all detected VOC features and time points. Plants with values outside the 1.5×IQR (interquartile range) were classified as outliers and excluded from the sensitivity analysis. Three plants met this criterion and were removed from the dataset.

The GAMM analyses to assess differences in VOCs intensity and temporal dynamics were then repeated on the reduced dataset using the same modeling structure, smoothing parameters, autocorrelation correction, and multiple-testing procedure adopted in the primary analyses.

The sensitivity analysis showed that the main condition-dependent VOCs differentiation patterns were broadly preserved after outlier removal. Specifically, 137 significant VOCs features overlapped between the original and reduced analyses (Jaccard index = 0.60), indicating substantial stability of the overall condition-dependent modulation patterns.

In contrast, support-dependent temporal dynamics showed greater sensitivity to individual-level variability, with 23 significant VOCs features overlapping between the original and reduced analyses (Jaccard index = 0.24). This higher sensitivity is consistent with the greater complexity and trajectory dependence of the temporal GAMM analyses.

Overall, these results support the robustness of the main condition-dependent VOCs differentiation patterns while indicating that the precise identification of temporally modulated VOCs features is more sensitive to inter-individual variability.
