# Supplementary material for: Metabolic signatures accompany exploratory movement in climbing plants
Source: PLoS One. 2026 Oct 30;21(10):e0361761. doi: 10.1371/journal.pone.0361761 (PMC13722212; doi:10.1371/journal.pone.0361761)
Supplement: Supplementary file 2 [file pone.0361761.s007.docx]

Reviewer #1

(1) Temper the claims regarding support perception versus direct evidence.

R1. We thank the Reviewer for this observation. We agree that the manuscript should not overstate support perception. We re-assessed the manuscript as a whole for formulations of this kind and revised one sentence in the Discussion where perception could be read as assumed rather than as an open question (L 460). Elsewhere the manuscript already presents the finding as an association: the title states that metabolic signatures accompany exploratory movement, and the closing paragraph of the Discussion states that our analyses reveal correlations rather than causal relationships.

(2) Discuss alternative cues, such as light, airflow, or humidity gradients.

R2. We thank the Reviewer for this helpful suggestion and agree that these alternative cues deserve explicit consideration. The Discussion considered light gradients, variation in VOC composition or concentration, and prior root-mediated interactions as potential non-mutually exclusive mechanisms. In response to the Reviewer’s comment, we have expanded this section to include airflow and humidity gradients (L 480–483).

(3) Clarify that movement–VOC relationships are correlational, not causal.

R3. We agree with the Reviewer that this distinction is essential and thank them for prompting us to make it clearer throughout the manuscript. Although the Discussion acknowledged that the observed relationships between VOC emissions and circumnutation are correlational, we recognized that the term “coupling” in the Results could be interpreted as implying a mechanistic relationship. We have therefore added a clarification at its first occurrence (L 416–418), specifying that “coupling” refers only to the statistical association between VOC intensity and a kinematic descriptor within the model and does not imply a causal or mechanistic link.

(4) Discuss the biological function and impact of the most significant VOCs.

R4. We have added a passage to the Discussion (L 518-523) addressing this point. Among the formulas with correspondence in previous studies on *Pisum sativum*, C_10_H_16_ corresponds to the monoterpenes, and we now note their contribution to defense against herbivores and pathogens, to responses to stress and to plant-plant signaling, and that their higher intensity in plants growing without a support is compatible with a broader shift in secondary metabolism accompanying exploratory growth. We have deliberately kept this interpretation at the level of compound class, since exact mass does not discriminate among isomers.

(5) Report effect sizes and variability alongside statistical significance.

R5. We thank the Reviewer for this suggestion. We agree that these quantities should also appear in the Results narrative alongside the significance counts. We have added two sentences in the Results (L 365-369 and L 427-432) to make this explicit. We also added a new panel C to Figure 5 displaying the magnitude of the between-condition difference for every detected feature across the analyzed mass range, so that effect sizes are now also represented graphically.

(6) Discuss whether VOC changes precede, coincide with, or follow climbing behavior.

R6. The study was designed specifically to characterize the phase preceding physical contact with the support: all analyses are restricted to the exploratory phase, before the tendrils reach and coil around the pole. In this sense the VOCs differences we report necessarily precede climbing behavior, as stated in the Discussion.

(7) Outline future studies on sensory and signaling mechanisms.

R7. We thank the Reviewer for this suggestion. We have expanded the section of the Discussion where we outline future studies to include the sensory and signaling mechanisms (L 511-513).

Reviewer #2

(1) As H1, H2, and H3 are currently framed, there appears to be conceptual overlap among the three hypotheses. Please simplify and clarify the distinctions between H1 and H2, and elaborate on all three to improve readability. The framing could emphasize the following differences: (i) which VOC features are present, (ii) how those VOCs change throughout circumnutation between the two conditions, and (iii) how strong the relationship is between these variables. Connect these with the findings in the results and discussion for more clarity and emphasis.

R1. We thank the Reviewer for this observation and the suggestions. We modified the original formulation to make the distinction between the three hypotheses more explicit. We have integrated the hypothesis statement in the Introduction to define each hypothesis by the comparison it makes: differences in magnitude (H1), differences in temporal dynamics (H2), and differences in metabolic-kinematic coupling (H3) (L 51-55). We also clarify that H1 and H2 are logically independent, since a feature may differ in average level without differing in trajectory shape, or vice versa (L 56-58). Finally, we made explicit connection to this three-level framing into the Results (L 326, L 101-103, L 347-349, L 356-358, L 372-373, L377, L380) and the Discussion (L 454-456, L 465, L 471, L 492), as suggested.

(2) The standard practice in MS is to use m/z with italic formatting. Please replace all instances of m/Q with m/z. Also, use superscripts for the numbers in the molecular formulae shown in Table 1.

R2. We thank the Reviewer for these formatting suggestions. All instances of m/Q have been replaced with italicized *m/z* throughout the manuscript (L 215, L 222, L 227, L 365, L 385 and Table 1). The numerals in the molecular formulae of Table 1 have been formatted as subscripts, in accordance with standard chemical nomenclature (C₁₀H₁₂O, C₁₀H₁₆, C₈H₁₀, C₇H₈, C₁₁H₁₆).

(3) Please show spectra or attach data for representative samples in which some of the differences described in the paper are indicated, even if not all spectral features have been annotated.

We have added a new panel to Figure 5 (panel C) showing a difference spectrum across the analyzed mass range. Each detected feature is positioned at its *m/z* reporting the difference in mean normalized signal between the two growth conditions, and is colored according to the outcome of the two statistical tests, using the same scheme as the volcano-style plots in panels A and B. In this way the differences described in the manuscript are indicated directly on the mass axis. The complete feature table, including detected masses, effect estimates and FDR-adjusted *p*-values for every feature, is provided as S1 Dataset. A sentence referring to the new panel has been added to the Results (L 363-365), and the caption of Figure 5 has been updated accordingly.

(4) For the PCA analysis presented in this paper, PCA may not be the best way to represent the data for this study because the classes to be differentiated were known a priori. Is there a reason why LDA was not considered a more suitable approach?

R4. PCA was chosen deliberately as an unsupervised method. Not using the class labels, any grouping visible in the component space reflects structure present in the data rather than separation imposed by the analysis. We considered this the more conservative choice for a descriptive figure. We agree, however, that a supervised analysis is warranted given that the classes are known a priori, and we have added it. As suggested by the reviewer we implemented a linear discriminant analysis and a random forest for comparison. Linear discriminant analysis was evaluated using stratified 5-fold cross-validation repeated 10 times. The two growth conditions were classified with an accuracy of 0.91 ± 0.07 (SD), against a majority-class baseline of 0.54. The random forest classifier was evaluated with the same procedure and gave 0.90 ± 0.09 (SD). Under random permutation of the condition labels both classifiers returned 0.50 (p < 0.01, 100 permutations). This additional supervised analysis is described in the Methods (L 244-253) and reported in the Results (L 339-343). The corresponding code has been updated to the deposited repository.

(5) Line 190: Please provide more details about the source conditions and ionization parameters.

R5. We thank the Reviewer for this request. We have expanded the description of the mass spectrometric measurement in the Methods (L 197-207) to include more details about the source and ionization conditions: the design of the AIM reactor and its interface to the TOF analyzer, the generation of the benzene reagent ions by VUV photoionization from a heated permeation tube, the permeation tube temperature (80 °C), the reagent flow (0.25 L/min), the reactor temperature (50 °C), the ionization source pressure (50 mbar) and the composition, concentration and flow of the standard mixture used for the weekly sensitivity and mass calibration checks. A full characterization of this ionization scheme is reported in a previous works which we now cite (Avesani et al. 2026). We also added another reference to a method paper describing the whole facility (Simonetti et al. 2026) that should provide a detailed overview of the system (L 97).
