# Supplementary material for: Metabolic signatures accompany exploratory movement in climbing plants
Source: PLoS One. 2026 Oct 30;21(10):e0361761. doi: 10.1371/journal.pone.0361761 (PMC13722212; doi:10.1371/journal.pone.0361761)
Supplement: Peer review history

# Peer review: Decision Letter 0

**DOI:** 10.1371/journal.pone.0361761.r001

**Authors:** Anil Bhatia

6 Sep 2026

Dear Dr.  Simonetti,

Thank you for submitting your manuscript to PLOS One. After careful consideration, we feel that it has merit but does not fully meet PLOS One’s publication criteria as it currently stands. Therefore, we invite you to submit a revised version of the manuscript that addresses the points raised during the review process.

Please submit your revised manuscript by Oct 06 2026 11:59PM. If you will need more time than this to complete your revisions, please reply to this message or contact the journal office at plosone@plos.org. When you're ready to submit your revision, log on to https://www.editorialmanager.com/pone/ and select the 'Submissions Needing Revision' folder to locate your manuscript file.

- A letter that responds to each point raised by the academic editor and reviewer(s). You should upload this letter as a separate file labeled 'Response to Reviewers'.
- A marked-up copy of your manuscript that highlights changes made to the original version. You should upload this as a separate file labeled 'Revised Manuscript with Track Changes'.
- An unmarked version of your revised paper without tracked changes. You should upload this as a separate file labeled 'Manuscript'.

If you would like to make changes to your financial disclosure, please include your updated statement in your cover letter. Guidelines for resubmitting your figure files are available below the reviewer comments at the end of this letter.

If applicable, we recommend that you deposit your laboratory protocols in protocols.io to enhance the reproducibility of your results. Protocols.io assigns your protocol its own identifier (DOI) so that it can be cited independently in the future. For instructions see: https://journals.plos.org/plosone/s/submission-guidelines#loc-laboratory-protocols. Additionally, PLOS One offers an option for publishing peer-reviewed Lab Protocol articles, which describe protocols hosted on protocols.io. Read more information on sharing protocols at https://plos.org/protocols?utm_medium=editorial-email&utm_source=authorletters&utm_campaign=protocols.

As the corresponding author, your ORCID iD is verified in the submission system and will appear in the published article. PLOS supports the use of ORCID, and we encourage all coauthors to register for an ORCID iD and use it as well. Please encourage your coauthors to verify their ORCID iD within the submission system before final acceptance, as unverified ORCID iDs will not appear in the published article. *Only*  the individual author can complete the verification step; PLOS staff *cannot*  verify ORCID iDs on behalf of authors.

We look forward to receiving your revised manuscript.

Kind regards,

Anil Bhatia, Ph.D

Academic Editor

PLOS One

Journal Requirements:

When submitting your revision, we need you to address these additional requirements.

1.Please ensure that your manuscript meets PLOS ONE's style requirements, including those for file naming. The PLOS ONE style templates can be found at

https://journals.plos.org/plosone/s/file?id=wjVg/PLOSOne_formatting_sample_main_body.pdf and

https://journals.plos.org/plosone/s/file?id=ba62/PLOSOne_formatting_sample_title_authors_affiliations.pdf

2. We note that the grant information you provided in the ‘Funding Information’ and ‘Financial Disclosure’ sections do not match.

When you resubmit, please ensure that you provide the correct grant numbers for the awards you received for your study in the ‘Funding Information’ section.

3. Thank you for stating the following financial disclosure:

“This research was funded by the European Union (ERC, ROOMors, Grant Number 101096728 to U.C.). Views and opinions expressed are however those of the author(s) only and do not necessarily reflect those of the European Union or the European Research Council Executive Agency. Neither the European Union nor the granting authority can be held responsible for them.”

Please state what role the funders took in the study.  If the funders had no role, please state: "The funders had no role in study design, data collection and analysis, decision to publish, or preparation of the manuscript."

If this statement is not correct you must amend it as needed.

Please include this amended Role of Funder statement in your cover letter; we will change the online submission form on your behalf.

4. Please note that your Data Availability Statement is currently missing the repository name and/or the DOI/accession number of each dataset OR a direct link to access each database. If your manuscript is accepted for publication, you will be asked to provide these details on a very short timeline. We therefore suggest that you provide this information now, though we will not hold up the peer review process if you are unable.

5. If the reviewer comments include a recommendation to cite specific previously published works, please review and evaluate these publications to determine whether they are relevant and should be cited. There is no requirement to cite these works unless the editor has indicated otherwise.

6. Please review your reference list to ensure that it is complete and correct. If you have cited papers that have been retracted, please include the rationale for doing so in the manuscript text, or remove these references and replace them with relevant current references. Any changes to the reference list should be mentioned in the rebuttal letter that accompanies your revised manuscript. If you need to cite a retracted article, indicate the article’s retracted status in the References list and also include a citation and full reference for the retraction notice.

[Note: HTML markup is below. Please do not edit.]

Reviewer's Responses to Questions

**Comments to the Author**

1. Is the manuscript technically sound, and do the data support the conclusions?

Reviewer #1: Yes

Reviewer #2: Yes

**********

2. Has the statistical analysis been performed appropriately and rigorously?

Reviewer #1: Yes

Reviewer #2: Yes

**********

3. Have the authors made all data underlying the findings in their manuscript fully available?

The PLOS Data policy

Reviewer #1: Yes

Reviewer #2: No

**********

4. Is the manuscript presented in an intelligible fashion and written in standard English?

Reviewer #1: Yes

Reviewer #2: Yes

**********

Reviewer #1: This manuscript presents an innovative and rigorous investigation of how plant movement and volatile organic compound (VOC) emissions are coordinated during climbing behavior in Pisum sativum. By combining high-resolution three-dimensional kinematic analysis with real-time PTR-TOF-MS, the authors establish a strong experimental framework to examine links between exploratory growth and metabolic responses. The study advances understanding of circum-nutation by showing that support availability is associated with distinct VOC profiles before physical contact. Its integration of plant physiology, movement ecology, and metabolomics is a major strength and offers a valuable contribution to the emerging field of plant behavior.

A key strength is the identification of treatment-dependent VOC signatures linked to exploratory tendril movement. Plants grown without support showed distinct VOC emissions compared with plants exposed to a climbing structure, with stronger correlations between VOC production and tendril kinematics. The association of many VOCs with tendril dynamics, rather than general growth alone, suggests physiological specialization during exploration. These findings provide compelling evidence that plant movement and metabolic activity are closely integrated and open important avenues for studying VOC-mediated environmental responses.

Overall, this is a well-designed and thought-provoking study addressing an important underexplored question in plant biology. The data quality, analytical approach, and interpretation are strong, and the conclusions are generally well supported. The manuscript provides novel insight into how climbing plants adjust physiological state in response to environmental context and should interest readers in plant physiology, chemical ecology, and behavioral biology. I strongly support publication of the manuscript in its current form, however, a revised version with the following minor comments would further strengthen it.

• Temper the claims regarding support perception versus direct evidence.

• Discuss alternative cues, such as light, airflow, or humidity gradients.

• Clarify that movement–VOC relationships are correlational, not causal.

• Discuss the biological function and impact of the most significant VOCs.

• Report effect sizes and variability alongside statistical significance.

• Discuss whether VOC changes precede, coincide with, or follow climbing behavior.

• Outline future studies on sensory and signaling mechanisms.

Reviewer #2: In this study, the authors sought to investigate whether VOCs released by climbing plants differ in the presence or absence of support. Overall, the study is well executed and uses a comprehensive methodology to examine the metabolic signature associated with a specific stimulus, namely the presence of support. This work could lay the groundwork for future studies of other stimuli and help advance the understanding of similar processes.

(1) As H1, H2, and H3 are currently framed, there appears to be conceptual overlap among the three hypotheses. Please simplify and clarify the distinctions between H1 and H2, and elaborate on all three to improve readability. The framing could emphasize the following differences: (i) which VOC features are present, (ii) how those VOCs change throughout circumnutation between the two conditions, and (iii) how strong the relationship is between these variables. Connect these with the findings in the results and discussion for more clarity and emphasis.

(2) The standard practice in MS is to use m/z with italic formatting. Please replace all instances of m/Q with m/z. Also, use superscripts for the numbers in the molecular formulae shown in Table 1.

(3) Please show spectra or attach data for representative samples in which some of the differences described in the paper are indicated, even if not all spectral features have been annotated.

(4) For the PCA analysis presented in this paper, PCA may not be the best way to represent the data for this study because the classes to be differentiated were known apriori. Is there a reason why LDA was not considered a more suitable approach?

(5) Line 190: Please provide more details about the source conditions and ionization parameters.

**********

what does this mean?). If published, this will include your full peer review and any attached files.

If you choose “no”, your identity will remain anonymous but your review may still be made public.

**Do you want your identity to be public for this peer review?**  For information about this choice, including consent withdrawal, please see our Privacy Policy

Reviewer #1: No

Reviewer #2: No

**********

[NOTE: If reviewer comments were submitted as an attachment file, they will be attached to this email and accessible via the submission site. Please log into your account, locate the manuscript record, and check for the action link "View Attachments". If this link does not appear, there are no attachment files.]

To ensure your figures meet our technical requirements, please review our figure guidelines: https://journals.plos.org/plosone/s/figures

You may also use PLOS’s free figure tool, NAAS, to help you prepare publication quality figures: https://journals.plos.org/plosone/s/figures#loc-tools-for-figure-preparation.

NAAS will assess whether your figures meet our technical requirements by comparing each figure against our figure specifications.

# Author comment: Author response to Decision Letter 1

**DOI:** 10.1371/journal.pone.0361761.r002

17 Sep 2026

Dear Editor and Reviewers,

we updated the funding information to include also the funder role. We also reported the data availability statement with the Zenodo repository in the manuscript.

All the reviewers' comments have been addressed and are available in the "Response to Reviewers" fileand we report them also here (references to lines refer to the Manuscript without track changes):

Reviewer #1

(1) Temper the claims regarding support perception versus direct evidence.

R1. We thank the Reviewer for this observation. We agree that the manuscript should not overstate support perception. We re-assessed the manuscript as a whole for formulations of this kind and revised one sentence in the Discussion where perception could be read as assumed rather than as an open question (L 460). Elsewhere the manuscript already presents the finding as an association: the title states that metabolic signatures accompany exploratory movement, and the closing paragraph of the Discussion states that our analyses reveal correlations rather than causal relationships.

(2) Discuss alternative cues, such as light, airflow, or humidity gradients.

R2. We thank the Reviewer for this helpful suggestion and agree that these alternative cues deserve explicit consideration. The Discussion considered light gradients, variation in VOC composition or concentration, and prior root-mediated interactions as potential non-mutually exclusive mechanisms. In response to the Reviewer’s comment, we have expanded this section to include airflow and humidity gradients (L 480–483).

(3) Clarify that movement–VOC relationships are correlational, not causal.

R3. We agree with the Reviewer that this distinction is essential and thank them for prompting us to make it clearer throughout the manuscript. Although the Discussion acknowledged that the observed relationships between VOC emissions and circumnutation are correlational, we recognized that the term “coupling” in the Results could be interpreted as implying a mechanistic relationship. We have therefore added a clarification at its first occurrence (L 416–418), specifying that “coupling” refers only to the statistical association between VOC intensity and a kinematic descriptor within the model and does not imply a causal or mechanistic link.

(4) Discuss the biological function and impact of the most significant VOCs.

R4. We have added a passage to the Discussion (L 518-523) addressing this point. Among the formulas with correspondence in previous studies on Pisum sativum, C10H16 corresponds to the monoterpenes, and we now note their contribution to defense against herbivores and pathogens, to responses to stress and to plant-plant signaling, and that their higher intensity in plants growing without a support is compatible with a broader shift in secondary metabolism accompanying exploratory growth. We have deliberately kept this interpretation at the level of compound class, since exact mass does not discriminate among isomers.

(5) Report effect sizes and variability alongside statistical significance.

R5. We thank the Reviewer for this suggestion. We agree that these quantities should also appear in the Results narrative alongside the significance counts. We have added two sentences in the Results (L 365-369 and L 427-432) to make this explicit. We also added a new panel C to Figure 5 displaying the magnitude of the between-condition difference for every detected feature across the analyzed mass range, so that effect sizes are now also represented graphically.

(6) Discuss whether VOC changes precede, coincide with, or follow climbing behavior.

R6. The study was designed specifically to characterize the phase preceding physical contact with the support: all analyses are restricted to the exploratory phase, before the tendrils reach and coil around the pole. In this sense the VOCs differences we report necessarily precede climbing behavior, as stated in the Discussion.

(7) Outline future studies on sensory and signaling mechanisms.

R7. We thank the Reviewer for this suggestion. We have expanded the section of the Discussion where we outline future studies to include the sensory and signaling mechanisms (L 511-513).

Reviewer #2

(1) As H1, H2, and H3 are currently framed, there appears to be conceptual overlap among the three hypotheses. Please simplify and clarify the distinctions between H1 and H2, and elaborate on all three to improve readability. The framing could emphasize the following differences: (i) which VOC features are present, (ii) how those VOCs change throughout circumnutation between the two conditions, and (iii) how strong the relationship is between these variables. Connect these with the findings in the results and discussion for more clarity and emphasis.

R1. We thank the Reviewer for this observation and the suggestions. We modified the original formulation to make the distinction between the three hypotheses more explicit. We have integrated the hypothesis statement in the Introduction to define each hypothesis by the comparison it makes: differences in magnitude (H1), differences in temporal dynamics (H2), and differences in metabolic-kinematic coupling (H3) (L 51-55). We also clarify that H1 and H2 are logically independent, since a feature may differ in average level without differing in trajectory shape, or vice versa (L 56-58). Finally, we made explicit connection to this three-level framing into the Results (L 326, L 101-103, L 347-349, L 356-358, L 372-373, L377, L380) and the Discussion (L 454-456, L 465, L 471, L 492), as suggested.

(2) The standard practice in MS is to use m/z with italic formatting. Please replace all instances of m/Q with m/z. Also, use superscripts for the numbers in the molecular formulae shown in Table 1.

R2. We thank the Reviewer for these formatting suggestions. All instances of m/Q have been replaced with italicized m/z throughout the manuscript (L 215, L 222, L 227, L 365, L 385 and Table 1). The numerals in the molecular formulae of Table 1 have been formatted as subscripts, in accordance with standard chemical nomenclature (C₁₀H₁₂O, C₁₀H₁₆, C₈H₁₀, C₇H₈, C₁₁H₁₆).

(3) Please show spectra or attach data for representative samples in which some of the differences described in the paper are indicated, even if not all spectral features have been annotated.

We have added a new panel to Figure 5 (panel C) showing a difference spectrum across the analyzed mass range. Each detected feature is positioned at its m/z reporting the difference in mean normalized signal between the two growth conditions, and is colored according to the outcome of the two statistical tests, using the same scheme as the volcano-style plots in panels A and B. In this way the differences described in the manuscript are indicated directly on the mass axis. The complete feature table, including detected masses, effect estimates and FDR-adjusted p-values for every feature, is provided as S1 Dataset. A sentence referring to the new panel has been added to the Results (L 363-365), and the caption of Figure 5 has been updated accordingly.

(4) For the PCA analysis presented in this paper, PCA may not be the best way to represent the data for this study because the classes to be differentiated were known a priori. Is there a reason why LDA was not considered a more suitable approach?

R4. PCA was chosen deliberately as an unsupervised method. Not using the class labels, any grouping visible in the component space reflects structure present in the data rather than separation imposed by the analysis. We considered this the more conservative choice for a descriptive figure. We agree, however, that a supervised analysis is warranted given that the classes are known a priori, and we have added it. As suggested by the reviewer we implemented a linear discriminant analysis and a random forest for comparison. Linear discriminant analysis was evaluated using stratified 5-fold cross-validation repeated 10 times. The two growth conditions were classified with an accuracy of 0.91 ± 0.07 (SD), against a majority-class baseline of 0.54. The random forest classifier was evaluated with the same procedure and gave 0.90 ± 0.09 (SD). Under random permutation of the condition labels both classifiers returned 0.50 (p < 0.01, 100 permutations). This additional supervised analysis is described in the Methods (L 244-253) and reported in the Results (L 339-343). The corresponding code has been updated to the deposited repository.

(5) Line 190: Please provide more details about the source conditions and ionization parameters.

R5. We thank the Reviewer for this request. We have expanded the description of the mass spectrometric measurement in the Methods (L 197-207) to include more details about the source and ionization conditions: the design of the AIM reactor and its interface to the TOF analyzer, the generation of the benzene reagent ions by VUV photoionization from a heated permeation tube, the permeation tube temperature (80 °C), the reagent flow (0.25 L/min), the reactor temperature (50 °C), the ionization source pressure (50 mbar) and the composition, concentration and flow of the standard mixture used for the weekly sensitivity and mass calibration checks. A full characterization of this ionization scheme is reported in a previous works which we now cite (Avesani et al. 2026). We also added another reference to a method paper describing the whole facility (Simonetti et al. 2026) that should provide a detailed overview of the system (L 97).

We thank you all for all your work

Attachment Submitted filename: Response to Reviewers.docx

# Peer review: Decision Letter 1

**DOI:** 10.1371/journal.pone.0361761.r003

**Authors:** Anil Bhatia

16 Oct 2026

<p>Metabolic signatures accompany exploratory movement in climbing plants

PONE-D-26-35889R1

Dear Dr. Simonetti,

We’re pleased to inform you that your manuscript has been judged scientifically suitable for publication and will be formally accepted for publication once it meets all outstanding technical requirements.

Within one week, you’ll receive an e-mail detailing the required amendments. When these have been addressed, you’ll receive a formal acceptance letter and your manuscript will be scheduled for publication.

An invoice will be generated when your article is formally accepted. Please note, if your institution has a publishing partnership with PLOS and your article meets the relevant criteria, all or part of your publication costs will be covered. Please make sure your user information is up-to-date by logging into Editorial Manager at Editorial Manager® and clicking the ‘Update My Information' link at the top of the page. For questions related to billing, please contact billing support.

If your institution or institutions have a press office, please notify them about your upcoming paper to help maximize its impact. If they’ll be preparing press materials, please inform our press team as soon as possible -- no later than 48 hours after receiving the formal acceptance. Your manuscript will remain under strict press embargo until 2 pm Eastern Time on the date of publication. For more information, please contact onepress@plos.org.

Kind regards,

Anil Bhatia, Ph.D

Academic Editor

PLOS One

Additional Editor Comments (optional):

Reviewers' comments:

Reviewer's Responses to Questions

**Comments to the Author**

Reviewer #1: All comments have been addressed

Reviewer #2: All comments have been addressed

**********

2. Is the manuscript technically sound, and do the data support the conclusions?

Reviewer #1: Yes

Reviewer #2: Yes

**********

3. Has the statistical analysis been performed appropriately and rigorously?

Reviewer #1: Yes

Reviewer #2: Yes

**********

4. Have the authors made all data underlying the findings in their manuscript fully available?

The PLOS Data policy

Reviewer #1: Yes

Reviewer #2: Yes

**********

5. Is the manuscript presented in an intelligible fashion and written in standard English?

Reviewer #1: Yes

Reviewer #2: Yes

**********

Reviewer #1: (No Response)

Reviewer #2: The manuscript has addressed the reviewer comments in a thorough and systematic manner. Hypotheses H1, H2, and H3 are now considerably clearer, and the authors have done an excellent job of clarifying the study hypotheses; the methods as well as the results and discussion sections are thorough. Adding LDA definitely strengthens the data package. Below are some recommendations which could strengthen the results and discussion section.

The manuscript should also temper claims regarding causality in the earlier portions. For example, in lines 74–78, the statement could be revised as follows: “These shifts highlight how environmental challenges may be associated with distinct metabolic responses that could form part of an adaptive strategy. This underscores the importance of environmental context in chemical signaling and offers new insights into how plants interact with their surroundings.” Wording such as “shaping anticipatory behavior” should be avoided unless the data directly support a causal interpretation. A nice addition could be mentioning which experiment could actually establish causality in the results and discussion.

The discussion would be strengthened by explaining how broadly the findings may be generalized and by outlining their wider implications. Because the study is strong (and points a future direction to include some confounding features), a clearer account of its relevance beyond the system examined would further enhance its impact.

The statement that circumnutation appears to depend on endogenous growth-regulating processes involving hormonal signaling and differential growth is interesting but requires clarification. The authors should clearly differentiate evidence-supported conclusions from proposed mechanisms and identify the additional studies required to assess causality while controlling for potential confounding factors especially here. They should also discuss the extent to which this interpretation may apply to other plant species.

**********

what does this mean?). If published, this will include your full peer review and any attached files.

If you choose “no”, your identity will remain anonymous but your review may still be made public.

**Do you want your identity to be public for this peer review?**  For information about this choice, including consent withdrawal, please see our Privacy Policy

Reviewer #1: No

Reviewer #2: No

**********

# Editor report: Acceptance letter

**DOI:** 10.1371/journal.pone.0361761.r004

**Authors:** Anil Bhatia

PONE-D-26-35889R1

PLOS One

Dear Dr. Simonetti,

I'm pleased to inform you that your manuscript has been deemed suitable for publication in PLOS One. Congratulations! Your manuscript is now being handed over to our production team.

At this stage, our production department will prepare your paper for publication. This includes ensuring the following:

* All references, tables, and figures are properly cited

* All relevant supporting information is included in the manuscript submission,

* There are no issues that prevent the paper from being properly typeset

You will receive further instructions from the production team, including instructions on how to review your proof when it is ready. Please keep in mind that we are working through a large volume of accepted articles, so please give us a few days to review your paper and let you know the next and final steps.

Lastly, if your institution or institutions have a press office, please let them know about your upcoming paper now to help maximize its impact. If they'll be preparing press materials, please inform our press team within the next 48 hours. Your manuscript will remain under strict press embargo until 2 pm Eastern Time on the date of publication. For more information, please contact onepress@plos.org.

You will receive an invoice from PLOS for your publication fee after your manuscript has reached the completed accept phase. If you receive an email requesting payment before acceptance or for any other service, this may be a phishing scheme. Learn how to identify phishing emails and protect your accounts at https://explore.plos.org/phishing.

If we can help with anything else, please email us at customercare@plos.org.

Thank you for submitting your work to PLOS One and supporting open access.

Kind regards,

PLOS One Editorial Office Staff

on behalf of

Dr. Anil Bhatia

Academic Editor

PLOS One
